# Host plant-mediated effects on *Buchnera* symbiont: implications for biological characteristics and nutritional metabolism of pea aphids (*Acyrthosiphon pisum*)

**DOI:** 10.3389/fpls.2023.1288997

**Published:** 2023-12-06

**Authors:** Hui-ping Liu, Qiao-yan Yang, Jing-xing Liu, Inzamam Ul Haq, Yan Li, Qiang-yan Zhang, Kotb A. Attia, Asmaa M. Abushady, Chang-zhong Liu, Ning Lv

**Affiliations:** ^1^ Biocontrol Engineering Laboratory of Crop Diseases and Pests of Gansu Province, College of Plant Protection, Gansu Agricultural University, Lanzhou, China; ^2^ Department of Biochemistry, College of Science, King Saud University, Riyadh, Saudi Arabia; ^3^ Biotechnology School, Nile University, 26th of July Corridor, Sheikh Zayed City, Giza, Egypt; ^4^ Department of Genetics, Agriculture College, Ain Shams University, Cairo, Egypt

**Keywords:** host plant, *Acyrthosiphon pisum*, primary symbiont, development and growth, nutrients

## Abstract

**Introduction:**

The pea aphid, *Acyrthosiphon pisum*, is a typical sap-feeding insect and an important worldwide pest. There is a primary symbiont-*Buchnera aphidicola*, which can synthesize and provide some essential nutrients for its host. At the same time, the hosts also can actively adjust the density of bacterial symbiosis to cope with the changes in environmental and physiological factors. However, it is still unclear how symbionts mediate the interaction between herbivorous insects’ nutrient metabolism and host plants.

**Methods:**

The current study has studied the effects of different host plants on the biological characteristics, *Buchnera* titer, and nutritional metabolism of pea aphids. This study investigated the influence of different host plants on biological characteristics, *Buchnera* titer, and nutritional metabolism of pea aphids.

**Results and discussion:**

The titer of *Buchnera* was significantly higher on *T. Pretense* and *M. officinalis*, and the relative expression levels were 1.966±0.104 and 1.621±0.167, respectively. The content of soluble sugar (53.46±1.97µg/mg), glycogen (1.12±0.07µg/mg) and total energy (1341.51±39.37µg/mg) of the pea aphid on *V. faba* were significantly higher and showed high fecundity (143.86±11.31) and weight (10.46±0.77µg/mg). The content of total lipids was higher on *P. sativum* and *T. pretense*, which were 2.82±0.03µg/mg and 2.92±0.07µg/mg, respectively. Correlation analysis found that the difference in *Buchnera* titer was positively correlated with the protein content in *M. officinalis* and the content of total energy in *T. pratense* (*P* < 0.05). This study confirmed that host plants not only affected the biological characteristics and nutritional metabolism of pea aphids but also regulated the symbiotic density, thus interfering with the nutritional function of *Buchnera*. The results can provide a theoretical basis for further studies on the influence of different host plants on the development of pea aphids and other insects.

## 1 Introduction

Symbiosis, a complex and mutually advantageous relationship between species, has consistently been a fundamental aspect of biological accomplishments (Vostinar et al., 2021). Within the expansive and heterogeneous realm of arthropods, a notable illustration of such cooperative associations can be observed in their interactions with microbes, specifically endosymbiotic bacteria (Perlmutter and Bordenstein, 2020). A remarkable finding that arises from current research is the prevalence and importance of symbiotic relationships in arthropods, with over 50% of arthropod species engaging in some partnership with symbionts (Weinert et al., 2015; Harumoto et al., 2016). One can witness the epitome of mutualistic relationships by delving further into the domain of Hemiptera, particularly the sub-order Sternorrhyncha, which encompasses psyllids, aphids, and mealybugs. These hemipteran insects engage in obligatory and facultative associations with various prokaryotic endosymbionts, rendering them a valuable resource for researchers seeking to comprehend the complexities of symbiotic partnerships (Douglas, 1998; Baumann, 2005).

The obligate symbionts, also known as primary symbionts, are notable for their essential function in the existence of their host insects. The role of these organisms extends beyond mere cohabitation, as they provide their hosts with vital nutrients, including amino acids and vitamins, which are typically lacking in their primary diet consisting of plant sap. The mutualistic association between insects and their endosymbiotic bacteria, such as the pea aphid-*Buchnera* and whitefly-*Portiera* models, has been extensively studied and well-documented in scientific literature. These studies have provided comprehensive insights into the essential functions performed by these bacteria in ensuring the survival of their host organisms (Sloan et al., 2014; Douglas, 2015; Morrow et al., 2017; Bao et al., 2021). At a more intricate molecular level, these obligate symbionts effectively enhance metabolic pathways within their hosts, thereby compensating for any nutritional deficiencies. The examination of this specific symbiotic alliance is of notable significance, as it provides insight into the mechanisms by which nature ensures survival in the face of dietary constraints (Wilson and Duncan, 2015; Bao et al., 2021; Zhu et al., 2022). One illustrative instance is the symbiotic relationship between *Francisella*, a microorganism, and the African soft tick, *Ornithodoros moubata*. This symbiosis is characterized by the production of vital molecules such as biotin and riboflavin by *Francisella*, which enhances the nutritional value of the tick’s diet. This example highlights the interdependence between the host organism and its symbiotic partner (Duron et al., 2018).

In contrast, secondary endosymbionts, while not indispensable for the viability of their hosts, play significant roles in influencing their ecological dynamics and behavioral patterns. The studies mentioned above by Lv et al. (2021) and Deehan et al. (2021) have demonstrated that microorganisms can exert an impact on dietary preferences, enhance defense mechanisms against predators and pathogens, and potentially induce modifications in reproductive strategies. Himler et al. (2011) reported an intriguing finding regarding Rickettsia-infected whiteflies, wherein the presence of the symbiont resulted in a range of advantages, including enhanced progeny production and a biased sex ratio favoring females. Moreover, the exceptional capabilities of secondary endosymbionts are demonstrated by their capacity to induce distinctive reproductive consequences, such as parthenogenesis and male killing. These intriguing phenomena have captured the interest of evolutionary biologists (Stouthamer et al., 1999; Werren et al., 2008).

The intricate interplay between hosts and their symbionts, characterized by genomic and metabolic integration, is a crucial aspect that should not be disregarded, as it has undergone significant refinement throughout evolutionary timescales (Moran et al., 2008; Zhao et al., 2020). The co-evolutionary trajectories and genome compatibility of the organisms under study are clearly indicative of a profound level of integration, as demonstrated by Wilson and Duncan (2015) and Moran and Bennett (2014). It is worth noting that the titers of endosymbionts within their respective hosts are not fixed entities. They exhibit dynamic adjustments in response to a wide range of external and internal factors. Various factors can influence the densities of endosymbionts, including the genetic composition of the host, environmental stressors, alterations in diet, and exposure to antibiotics (Cassone et al., 2015; Zhang et al., 2016; Nguyen et al., 2017; Zhang et al., 2019; Bulman et al., 2021). As an illustration, the investigation conducted by Patton et al. (2021) revealed that specific viral infections in aphids resulted in a significant decrease in the densities of their *Buchnera* symbionts. In addition, the authors of Funkhouser-Jones et al. (2018) have identified specific host genes that are directly involved in the regulation of symbiont densities, thereby adding further intricacy to this association. The involvement of cellular machinery, particularly the autophagy mechanism, has been proposed as a significant factor in regulating these interactions, indicating a potentially fruitful avenue for further investigation (Cassone et al., 2015; Wang et al., 2022).

The pea aphid *Acyrthosiphon pisum* (Hemiptera: Aphididae) is a major economic pest in agriculture and forestry worldwide, known for consuming a wide range of legume plants. The pea aphid is a typical insect with alternating generations, which has the characteristics of rapid parthenogenic reproduction and a short life cycle. It not only causes discoloration, curl and deformity of plant leaves by feeding the phloem sap of host plants but also affects the normal growth and development of plants, resulting in a decline in yield and quality (Losey and Eubanks, 2000; Ryalls et al, 2013). It has the ability to transmit more than 30 plant viruses, resulting in substantial agricultural damage (Golawska and Lukasik, 2012). It is worth noting that pea aphids feed on the phloem sap of host plants, and there are limited amounts of essential amino acids and vitamins in the plant sap (Karley et al, 2002; Douglas, 2006). The core component of their biological makeup is the principal endosymbiont, *Buchnera aphidicola*. The symbiotic relationship between the aphid and its symbiont is characterized by providing a diverse range of vital nutrients that enhance the aphid’s dietary requirements. The initial investigations have demonstrated the negative impact on the reproductive capacity and development of aphids when there are disturbances in this mutually beneficial association (Lv et al., 2018). Nevertheless, the intricate relationships among these symbiotic organisms, herbivorous insects, and their host plants remain poorly understood. The current study aims to thoroughly examine the symbiotic interactions between pea aphids and *Buchnera*, focusing on their dynamics under varying host plant conditions. By comprehensively evaluating factors such as aphid weight, fecundity, *Buchnera* densities, and nutrient content, we aim to understand the influence of different host plants on the development of pea aphids and the inherent complexity of these interrelationships and to understand the different phenotypes of aphids that may be produced under the influence of different host plants. This comprehension can provide a theoretical basis for further studies on the effects of different host plants on the development of pea aphids and other insects, and provide strong support for clarifying the population evolution and species formation of aphids, and possesses the potential to illuminate the intricacies of symbiotic associations and their wider ramifications for the dynamics of ecosystems.

## 2 Materials and methods

### 2.1 Insects

The green color morphs of the pea aphid, *A. pisum*, were separately collected from alfalfa fields in Lanzhou City, Gansu Province, China. A single asexual line was established for further laboratory experiments by parthenogenesis of one pea aphid. The main host plant utilized for rearing pea aphids in our study was broad bean (*Vicia faba*). To establish the experimental population of pea aphids, pea aphids were maintained in the laboratory at a controlled temperature of 22 ± 1 °C and relative humidity of 70-80%, with a photoperiod of 16 hours of light and 8 hours of darkness (16:8 L:D). The aphids were reared on the broad bean plants for a minimum of three generations before being used in the experiments.

### 2.2 Host plant

In the current study, a total of six different host plant species were utilized for the experimental procedures. Expanding upon the cultivation methodologies outlined by Lv et al. (2018), the seedlings were carefully nurtured in a controlled environment. The broad bean (*V. faba*) was selected as the primary host for this research, as it provided a suitable environment for breeding our experimental pea aphid population. Furthermore, distinct populations of pea aphids were created with in a controlled laboratory setting by utilizing five different plant species, namely pea (*Pisum sativum*), alfalfa (*Medicago sativa*), clover (*Trifolium pratense*), red bean grass (*Onobrychis viciaefolia*), and melilotus (*Melilotus officinalis*). The aphid colonies were cultivated on their designated host plants for a minimum of three generations prior to their inclusion in our experiments. The host plants exhibited robust growth within a controlled greenhouse environment, where precise conditions were upheld. These conditions included a temperature range of 22 ± 1 °C, relative humidity levels ranging from 70% to 80%, and a light-dark cycle of 16 hours of light followed by 8 hours of darkness. The plants that were selected for further laboratory experiments were only those that demonstrated the highest level of resilience and durability.

### 2.3 Biological assay of pea aphids’ weight and fecundity

The present study utilized bioassays to evaluate the influence of different host plants on the weight and reproductive capacity of pea aphids. Aphids are raised by the detached leaves-feeding method; the experiment was initiated by preparing a Petri dish with a diameter of 10 cm and then lining it with filter paper. Subsequently, a leaf in a pristine condition, positioned with its blade oriented in an upward direction, was meticulously inserted into the dish. The petioles were carefully wrapped with absorbent cotton balls that had been dampened with distilled water (ddH_2_O) to ensure sufficient moisture for both the cotton balls and the filter paper. Following this, a recently hatched aphid, with age not exceeding 6 hours, was introduced into the Petri dish, enabling it to consume the leaf obtained from one of the previously mentioned six plant species. The aphids were placed individually in Petri dishes and placed in a controlled environment chamber (RZX, Ningbo Jiangnan Co. Ltd., Ningbo, China). The chamber was set to maintain a temperature of 22 ± 1 °C, a relative humidity of 70-80%, and a photoperiod of 16:8 (L:D). The aged leaves were replaced with newly harvested counterparts on a tri-daily basis. A sample size of 60 aphids was utilized for each host plant. Systematic monitoring of aphid populations on various host plants was conducted. The researchers meticulously recorded data about mortality and molting patterns, including the frequency and timing of molting, at regular 12-hour intervals. To facilitate accurate observations, the molting dander is carefully selected and collected with a camel brush to ensure precise data collection without disturbing the aphids or their environment. When the aphids developed into an adult (after the fourth molting), ten pea aphids from each host plant were randomly selected and weighed using a high-precision (1/100,000) electronic balance. Five individuals were randomly selected from each host plant to quantify *Buchnera* in the pea aphid. In addition, five pea aphids were randomly selected from the same host plant for quantitative analysis of nutrients, including total lipid, total protein, soluble sugar, and glycogen. These experiments were repeated three times. Thirty aphids were randomly selected from the same host plant to determine their reproductive capacity. Observe and record the number of nymphs produced by each aphid on a daily basis until the adult aphids died. According to the observed data, the fecundity of pea aphid on each of the six host plants was calculated.

### 2.4 Quantitative detection of *Buchnera*

The quantification of *Buchnera* presence in pea aphids across different host plants was conducted through the utilization of quantitative real-time PCR (q-PCR). Deoxyribonucleic acid extraction was performed on clusters of five recently matured adult aphids for each sample in this study. The Direct-zol RNA Kits (Zymo Research, Irvine, CA, USA) were used for the isolation process. In previous studies conducted by Zhang et al. (2016) and Chang et al. (2022), the *Buchnera* 16S rRNA gene was utilized as the detection target, while the ef1α gene of the aphid was selected as the reference gene. This choice of reference gene enabled the normalization and quantification of data, as described in the studies as mentioned earlier. Detailed information on primer sequences is included in **Supplementary File S1**. The quantitative polymerase chain reaction (qPCR) was performed using SYBR premix Ex Taq (TaKaRa, Japan) and conducted on an ABI 7500 Real-Time PCR Detection System (Applied Biosystems/Life Technologies, USA). The qPCR mixture consisted of 5 μL of SYBR Green PCR Mastermix (TaKaRa), 1 μL of the DNA extract, 1 μL (10 μM) of the forward primer, 1 μL (10 μM) of the reverse primer, and 3 μL of sterile distilled water, resulting in a total volume of 10 μL. The thermocycling protocol consisted of an initial denaturation step at a temperature of 95°C for a duration of 30 seconds, followed by 40 cycles of denaturation at 95°C for 5 seconds, and subsequent annealing/extension at a temperature of 60°C for a duration of 34 seconds. Subsequently, a melt curve analysis was conducted, commencing at a temperature of 95°C for a duration of 15 seconds, followed by a decrease to 60°C for a period of 1 minute, and ultimately concluding at a temperature of 95°C. The experimental protocol was replicated four times.

### 2.5 Samples preparation for nutrient determination

A total of 90 adult aphids were collected from six different host plants to evaluate their nutritional composition, including components such as total protein, soluble sugar, glycogen, and total lipid. In order to provide further clarification regarding the methodology, a total of five mature aphids were selected from each host plant. These aphids were subjected to a thorough rinse using ddH_2_O before being carefully transferred into a 1.5 mL centrifuge tube containing 180 µL of an aqueous lysis buffer formulation. The buffer solution consisted of a concentration of 100 millimolar (mM) potassium dihydrogen phosphate (KH_2_PO_4_), 1 mM ethylenediaminetetraacetic acid (EDTA), and 1 mM dithiothreitol (DTT). The pH of the buffer was maintained at 7.4. Subsequently, a comprehensive standardization of the aphids was performed. The resultant mixture was subjected to centrifugation at a velocity of 16,000 revolutions per minute (rpm), sustained for a duration of 15 minutes at a temperature of 4 degrees Celsius. The liquid portion obtained from this procedure was subsequently designated for subsequent nutritional evaluations. The entire procedure was repeated three times for each host plant, and the technique was replicated twice for each biological sample.

### 2.6 Protein content detection

The protein concentration in the pea aphid was determined using the methodologies described by Li et al. (2021) and Lv et al. (2018). Approximately 20 µL of the homogenized sample from the aphid was carefully transferred into a 1.5 mL centrifuge tube using a pipette. Subsequently, a mixture of 200 µL of coomassie brilliant blue G-250 was combined with the aforementioned sample and left to incubate for a duration of 15 minutes at room temperature. After the completion of the incubation period, the resultant mixture was introduced into a 96-well borosilicate microplate. Subsequently, spectrophotometric measurements were conducted at a wavelength of 595 nm in order to assess the protein concentration. To establish a basis for these measurements, bovine serum albumin (BSA) was dissolved in a buffer of identical composition and subsequently diluted in a stepwise manner to generate a range of concentrations. This diluted BSA solution served as a calibration standard for the experiment. The total protein content of the aphid samples was determined by employing the established curve derived from bovine serum albumin obtained from Sangon Biotech in Shanghai, China. The investigative procedure was repeated six times.

### 2.7 Sample preparation for the determination of soluble sugar, glycogen content, and total lipid

A volume of 180 µL of the homogenate supernatant was transferred to a new centrifuge tube with a capacity of 2 mL. A 20 µL aliquot of a 20% sodium sulfate solution was introduced to facilitate the dissolution of total carbohydrates. In order to enhance the solubility of both the total lipid and water-soluble carbohydrates, the solution was supplemented with 1,500 µL of a chloroform-methanol mixture in a 1:2 volume ratio. The composite sample was centrifugated at a speed of 10,000 revolutions per minute for 15 minutes at a temperature of 4°C. After centrifugation, the translucent liquid above the sediment was meticulously transferred using a pipette into a separate centrifuge tube. This particular tube was designated for future analysis of the overall lipid content and soluble sugar concentration. In contrast, the sediment that remained at the bottom of the tube was set aside to analyze its glycogen content.

### 2.8 Total lipids content detection

The methodology for determining the total lipid content in pea aphid followed procedures delineated by Handel (1965) and Lv et al. (2018). A volume of 100 µL of the previously mentioned supernatant was transferred into a centrifuge tube with a capacity of 1 mL using a pipette. The provided specimen was subsequently exposed to a temperature of 90 °C until the solvent was completely evaporated. Following that, a volume of 10 µL of concentrated sulfuric acid (98%) was added to the tube. After the inclusion of this component, the tube was subjected to incubation at a temperature of 90 °C for a short duration of 2 minutes, and subsequently rapidly cooled using ice. A 190 µL aliquot of a vanillin solution, which was prepared at a concentration of 1.2 g/L using 68% orthophosphoric acid as the solvent, was introduced into the cooled tube. The reaction was allowed to proceed for a period of 15 minutes at room temperature. The resulting mixture was subsequently distributed into a 96-well borosilicate microplate. Spectrophotometric measurements were conducted to assess the optical density (OD) values at a specific wavelength of 525 nm. The overall lipid content was determined by utilizing the standard curve derived from triolein. The experiment was systematically replicated on six occasions.

### 2.9 Soluble sugar content detection

The soluble sugar concentration in pea aphids was quantified according to the methodologies described by Handel (1985) and Handel and Day (1988). In summary, a volume of approximately 150 µL of the supernatant obtained from each aphid specimen was transferred into a 1.5 mL centrifuge tube and subjected to complete evaporation under normal atmospheric conditions. Following that, a volume of 10 µL of distilled water was mixed with 240 µL of an anthrone reagent solution (with a concentration of 1.42 g/L) that had been prepared using 70% sulphuric acid. The mixture contained in the tube was allowed to incubate at ambient temperature for a duration of 15 minutes, subsequently followed by a 15-minute immersion in a boiling water bath. Following the post-heating process, the tubes were subsequently brought back to ambient temperature to facilitate the cooling process. Subsequently, the resulting mixture was transferred to a microplate made of borosilicate glass with 96 wells. Spectrophotometric measurements were conducted to obtain optical density (OD) readings at a wavelength of 630 nm. Soluble sugar concentrations were determined using a reference standard curve prepared with D-glucose. The aforementioned analytical procedure was performed on six separate occasions consistently.

### 2.10 Glycogen content detection

The glycogen content of the pea aphid was detected according to the method of Lv et al. (2018) and Li et al. (2021). The precipitant from pre-preparation was mixed with 400 µL of 80% methanol and made turbid by sonicating in an ultrasonic cleaning for 10 min, after which the homogenate was centrifuged again at 10,000 rpm for 5 min at 4 °C, and the supernatant was removed with a 1.5 mL centrifuge tube. Next, 1,000 µL of 1.42 g/L anthrone reagent (solvent was sulphuric acid 70%) were added to the precipitant, and the tube was incubated for 15 min at room temperature and then incubated in boiling water for a further 15 min, followed by cooling on ice to stop the reaction. After this mixture was transferred into a 96-well borosilicate microplate, the absorbance value was measured spectrophotometrically at 630 nm wavelength. As the standard, D-glucose was used to calculate the soluble sugar content. The experiment was repeated six times. The different nutrients (fats, proteins, soluble sugar, and glycogen) have been converted to energy equivalents. These reserves have energy equivalents of 39,500 mJ/mg for lipids, 24,000 mJ/mg for proteins, and 17,500 mJ/mg for soluble sugar and glycogen.

### 2.11 Statistical analyses

The data were organized and sorted utilizing Microsoft Excel version 2021. GraphPad Prism 8, a software provided by Systat Software Inc. in San Jose, CA, USA, was utilized to create subsequent graphical representations. In order to conduct statistical analyses, the researchers utilized IBM SPSS Statistics version 22.0 (IBM, Armonk, NY, USA). In order to identify noteworthy differences among different treatments, the statistical technique of analysis of variance (ANOVA) was employed, specifically utilizing Tukey’s honestly significant difference (HSD) test.

## 3 Results

### 3.1 Effects of different host plants on the weight of pea aphid

**Figure 1:**
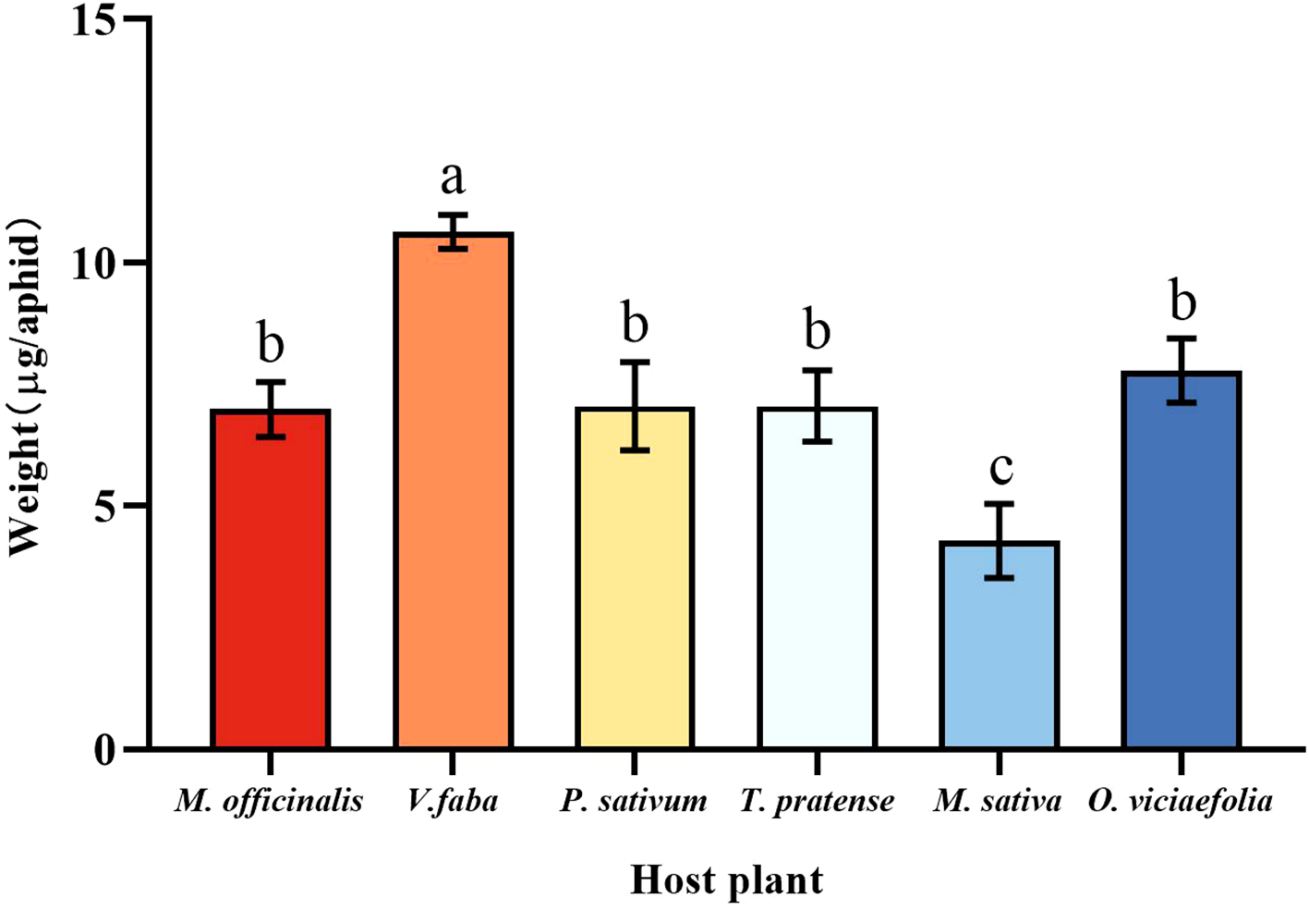
Effects of different host plants on the weight of pea aphids. Different lowercase letters between different host plants are significant differences as determined by one-way ANOVA Tukey’s HSD test at *p* < 0.05.

The six host plants affect the weight of the pea aphid differently. Compared with other host plants, the weight of a pea aphid fed on *V. faba* was the highest, and the weight of a pea aphid fed on *M. sativa* was the lowest. However, there were no significant differences in weight that fed on *M. officinalis*, *T. pratense*, *P. sativum*, and *O. viciaefolia* (**Figure 1**, *F* (5, 174) = 43.185, *P* < 0.001). This indicates that pea aphid has good adaptability on *V. faba.*

### 3.2 The fecundity of pea aphids on different host plants

**Figure 2:**
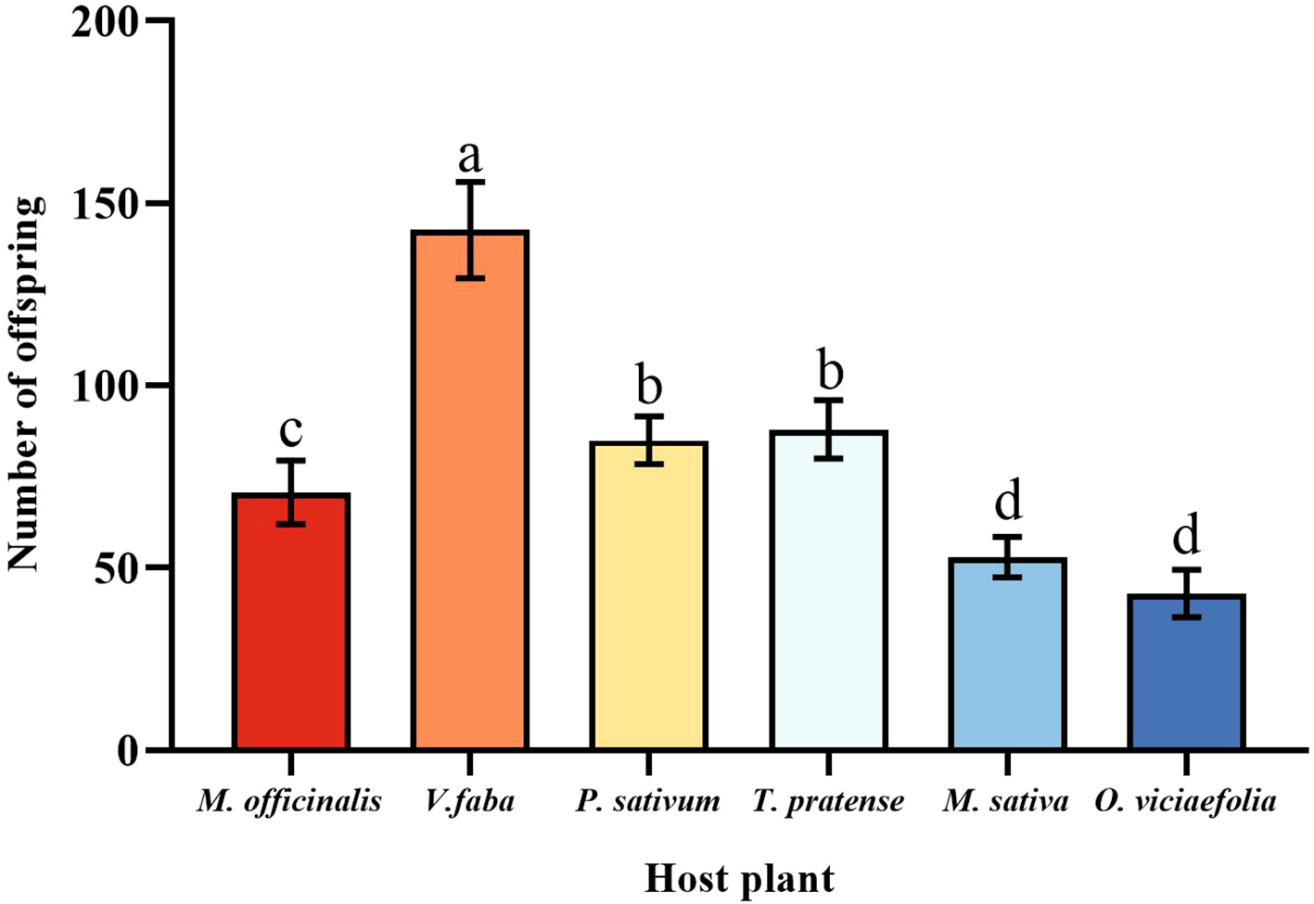
Effects of different host plants on the fecundity of pea aphids. Different lowercase letters between different host plants are significant differences as determined by one-way ANOVA Tukey’s HSD test at *p* < 0.05.

Pea aphids have different fecundity on different host plants; the pea aphid had the highest fecundity fed on *V. faba* in six host plants, which was a significant difference compared to other host plants. The fecundity of pea aphids on *O. viciaefolia* was the lowest in six host plants, but pea aphis showed no significant difference from *M. sativa*. The fecundity of pea aphids on *M. officinalis* significantly differed from the other host plants. In addition, there was no significant difference in the fecundity of pea aphids between *P. sativum* and *T. pratense*. The data showed that among pea aphids, feeding on *V. faba* is beneficial to the fecundity of aphids. (**Figure 2**, *F* (5, 174) = 43.185, *P* < 0.001).

### 3.3 Titer mensuration of *Buchnera* in the pea aphid

**Figure 3:**
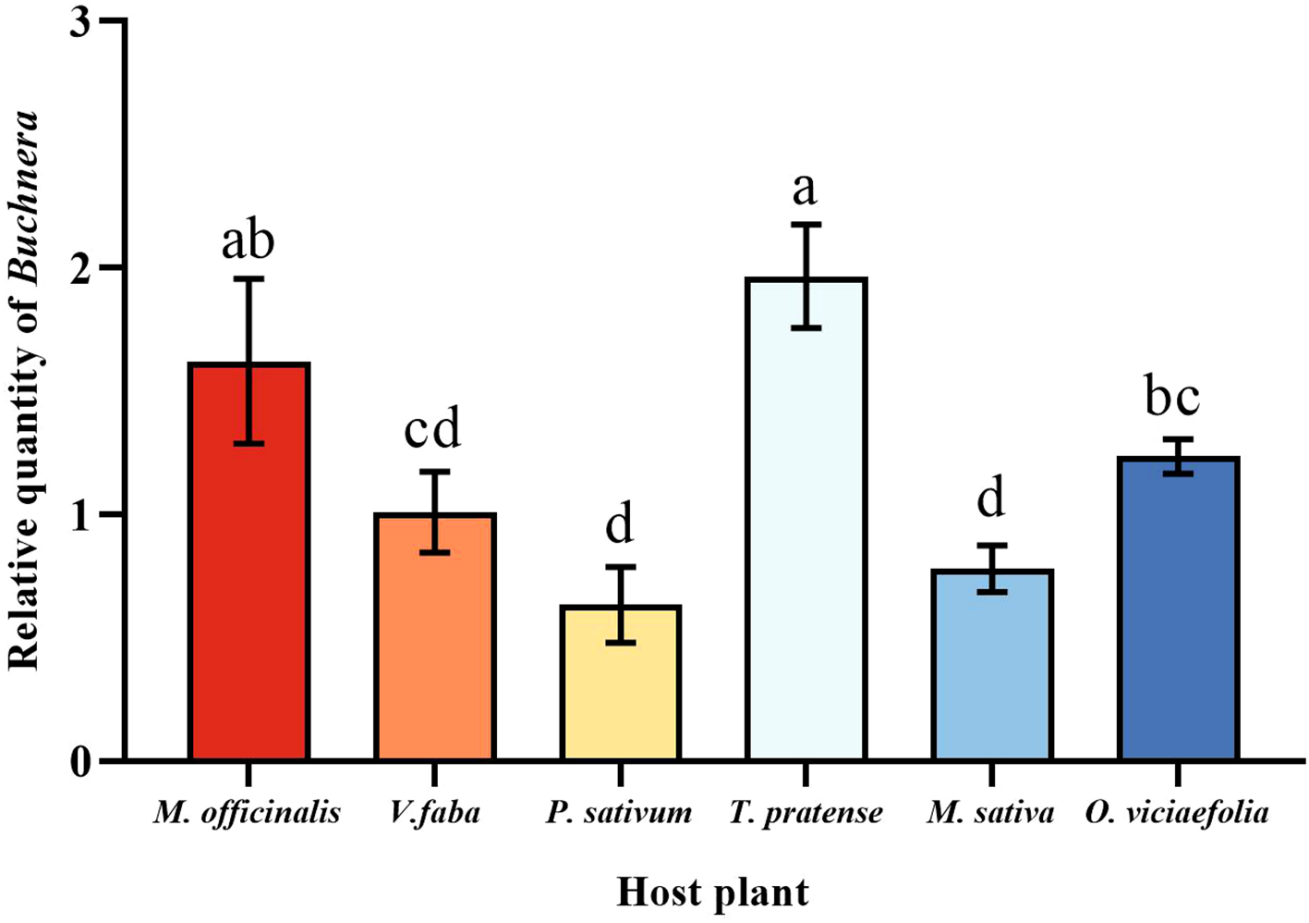
Effects of different host plants on the *Buchnera* titer in pea aphids. Different lowercase letters between different host plants are significant differences as determined by one-way ANOVA Tukey’s HSD test at *p* < 0.05.

The relative expression of *Buchnera* in the pea aphid was variable depending on the host plant. The relative expression of *Buchnera* in pea aphids fed on *T. pratense* was significantly higher than that of *V. faba*, *P. sativum*, *M. officinalis*, and *O. viciaefolia*. The relative expression of *Buchnera* in pea aphids was significantly different in the *V. faba* and *O. viciaefolia* compared with other host plants, but not significantly from each other (**Figure 3**, *F* (5, 18) = 29.873, *P* < 0.001). The results showed that host plants significantly affected the titer of symbiotic bacteria in pea aphids, which may be related to the nutritional status of host plants.

### 3.4 The protein content of different host plants of pea aphids

**Figure 4:**
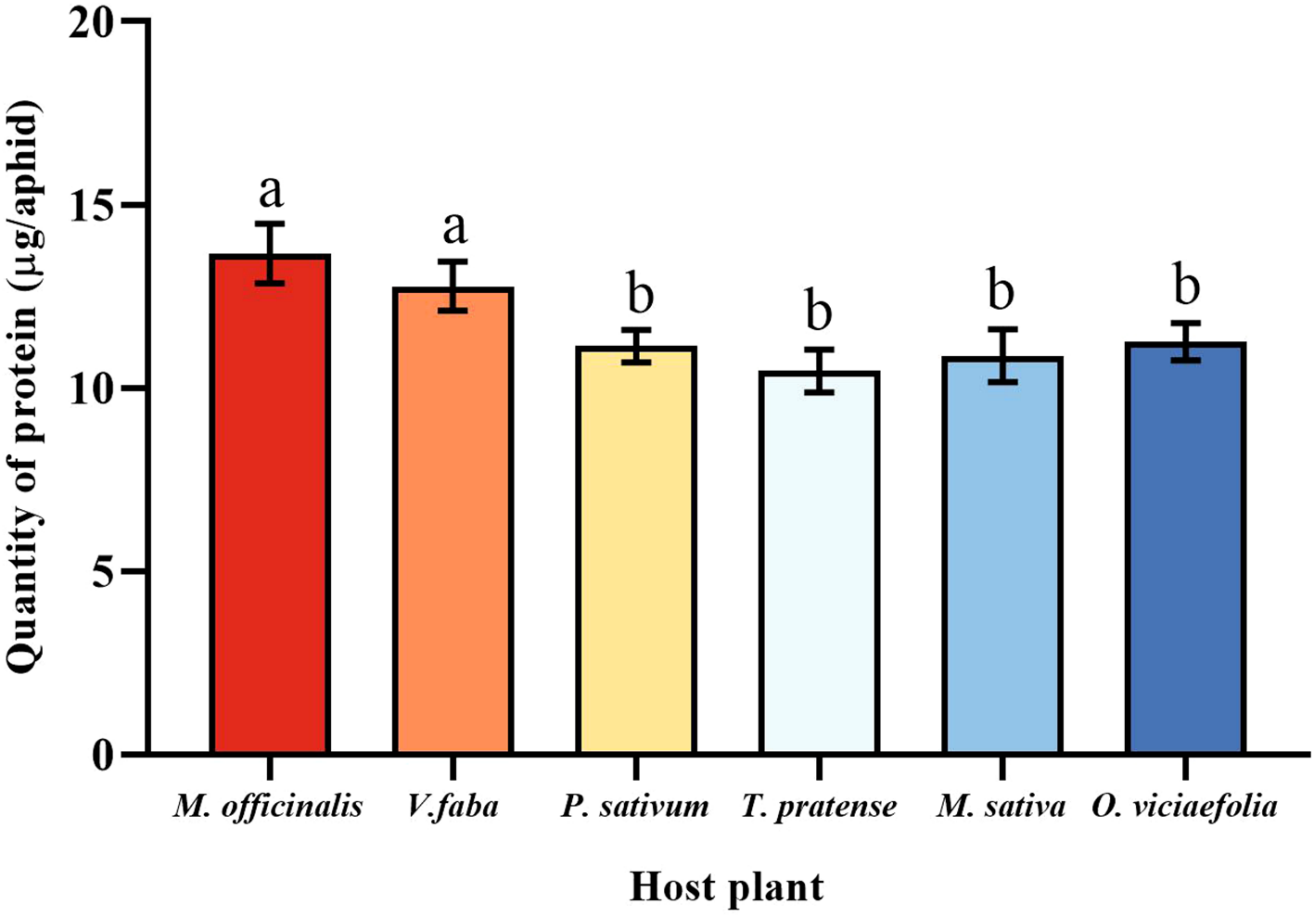
Effects of different host plants on the protein quantity of pea aphid. Different lowercase letters between different host plants are significant differences as determined by one-way ANOVA Tukey’s HSD test at *p* < 0.05.

The total protein content of pea aphids was different fed on the different host plants. The total protein content of pea aphids was significantly different in the *M. officinalis* and *V. faba* compared with other host plants, but not significantly different from each other. However, the total protein content of pea aphid was not significantly different among the *T. pratense*, *P. sativum*, *O. viciaefolia*, and *M. sativa*. (**Figure 4**, *F* (5, 30) = 8.611, *P* < 0.001). It can be seen that feeding on *M. officinalis* and *V. faba* improves the protein content in pea aphids.

### 3.5 Quantification of different host plants of pea aphids to soluble sugar

**Figure 5:**
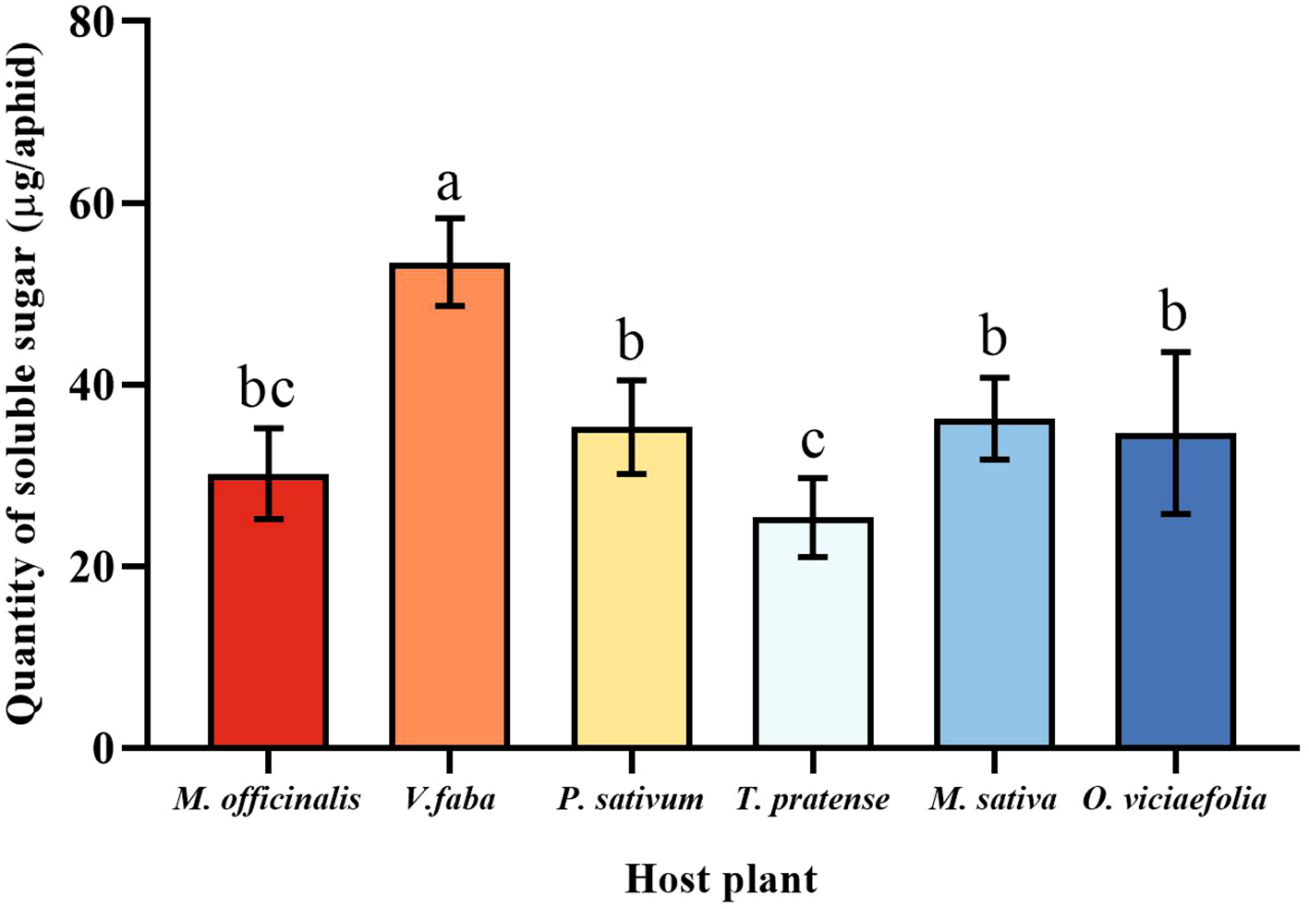
Effects of different host plants on the soluble sugar quantity of pea aphids. Different lowercase letters between different host plants are significant differences as determined by one-way ANOVA Tukey’s HSD test at *p* < 0.05.

The soluble sugar level of pea aphids varied among the six host plants. The maximum quantity of soluble sugar of pea aphid was fed on *V. faba*, which differed statistically from other plants. The soluble sugar content of the pea aphid fed on *T. pratense* was the lowest, which is not statistically different from *M. officinalis*. Moreover, the soluble sugar content of the pea aphids among *P. sativum*, *M. officinalis*, *M. sativa*, and *O. viciaefolia* showed no significant differences, which indicated that the soluble sugar content in the pea aphid is higher after feeding on *V. faba*. (**Figure 5**, *F* (5, 30) = 16.896, *P* < 0.001).

### 3.6 Glycogen content of different host plants of the pea aphid

**Figure 6:**
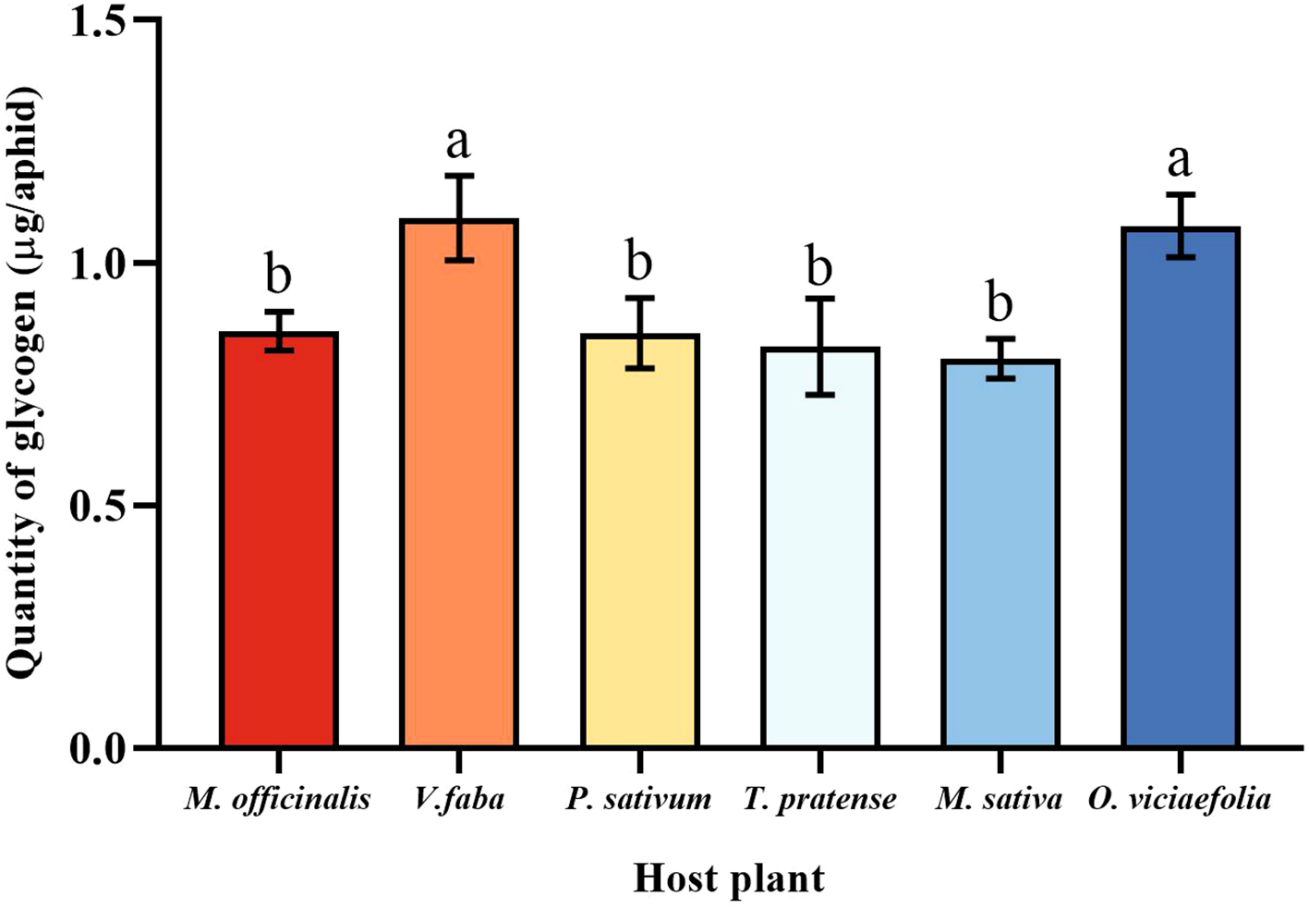
Effects of different host plants on the glycogen quantity of pea aphids. Different lowercase letters between different host plants are significant differences as determined by one-way ANOVA Tukey’s HSD test at *p* < 0.05.

Glycogen and soluble sugar are important carbohydrates in aphids, the different host plants of the pea aphid have different patterns in the content of the two carbohydrates. The glycogen of pea aphids fed on *V. faba* and *O. viciaefolia* was significantly higher than that of the other host plants, but the differences between them were insignificant. There were no significant differences in glycogen of pea aphids among *P. sativum*, *M. officinalis*, *M. sativa*, and *T. pratense* had no significant differences (**Figure 6**, *F* (5, 30) = 6.233, *P* < 0.001), suggesting that feeding on *V. faba* and *O. viciaefolia* was conducive to the increase of glycogen in pea aphids.

### 3.7 Quantification of different host plants of pea aphids to total lipids

**Figure 7:**
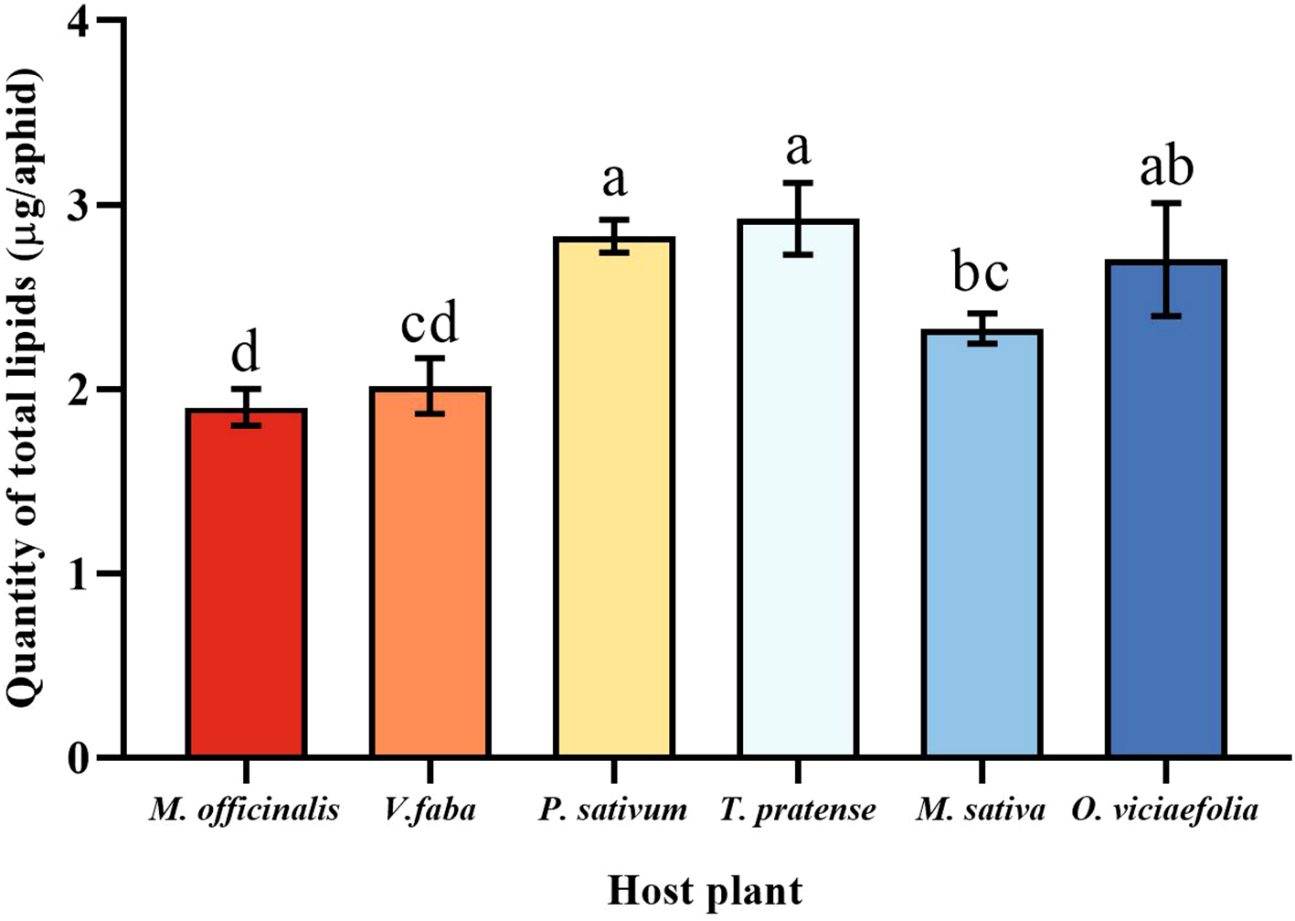
Effects of different host plants on the total lipid quantity of pea aphid. Different lowercase letters between different host plants are significant differences as determined by one-way ANOVA Tukey’s HSD test at *p* < 0.05.

The total lipids content of the pea aphid was variable depending on the host plant. The total lipids content of the pea aphid fed on *T. pratense* and *P. sativum* were significantly greater than that of the *V. faba*, *M. officinalis*, and *M. sativa*. The total lipids content of pea aphid was significantly different in the *M. officinalis* and *V. faba* compared with other host plants, but not significantly different from each other (**Figure 7**, *F* (5, 30) = 10.545, *P* < 0.001).

### 3.8 Effects of different host plants on the total energy of pea aphid

**Figure 8:**
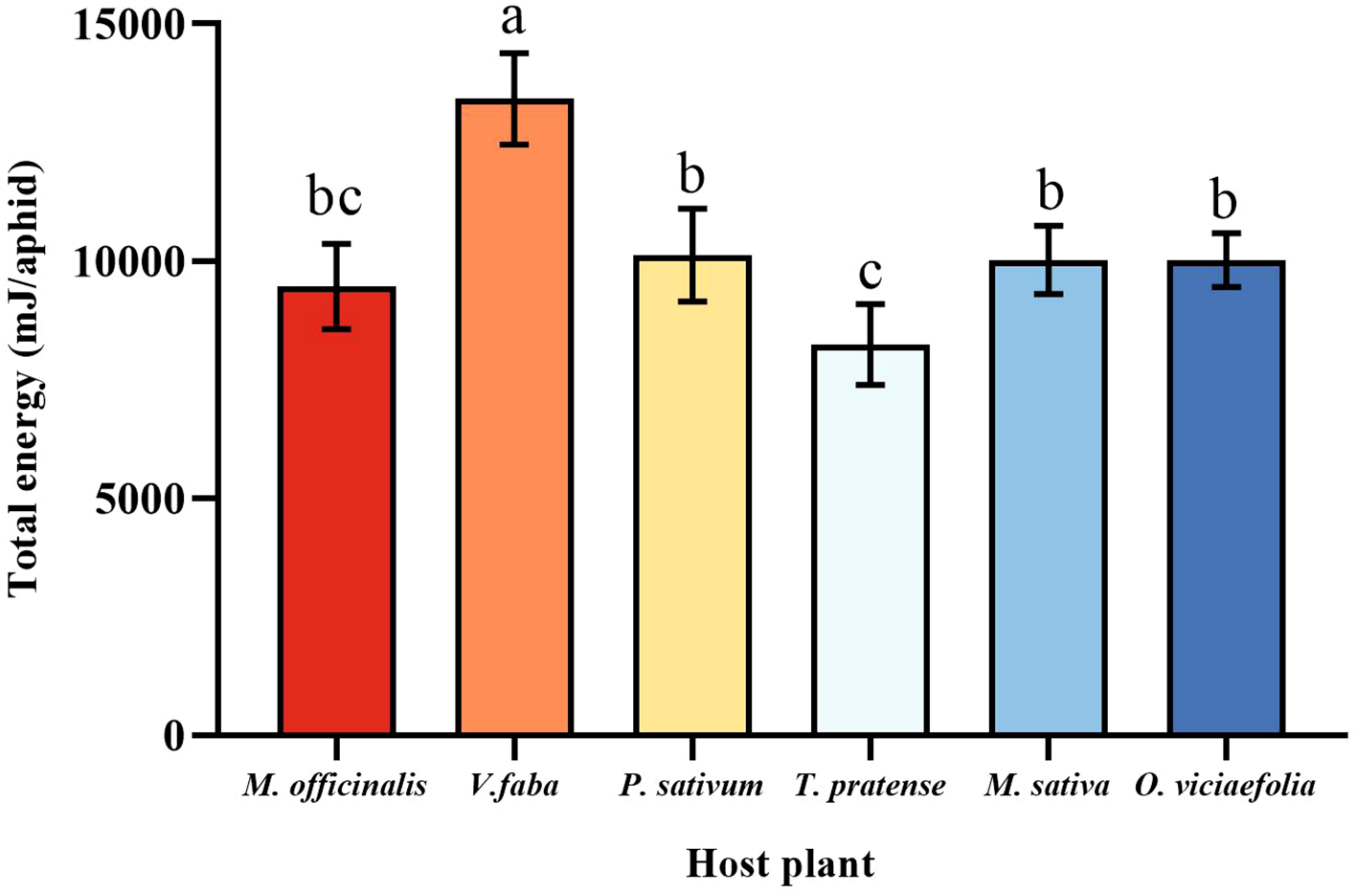
Effects of different host plants on the total energy of pea aphid. Different lowercase letters between different host plants are significant differences as determined by one-way ANOVA Tukey’s HSD test at *p* < 0.05.

The maximum total energy of pea aphid was fed on *V. faba*, which differed statistically from other plants. The total energy of the pea aphid fed on *T. pratense* was the lowest, which is not statistically different from *M. officinalis*. Furthermore, the total energy of the pea aphids among *P. sativum*, *M. officinalis*, *M. sativa*, and *O. viciaefolia* showed no significant differences (**Figure 8**, *F* (5, 30) = 16.328, *P* < 0.001).

### 3.9 Correlation between *Buchnera* titer and biological characteristics and nutrient metabolism of the pea aphid

Correlation analysis found that the *Buchnera* titer of pea aphids fed on different host plants had different correlations with biological characteristics and nutritional metabolism (**Table 1**). The difference in *Buchnera* titer was positively correlated with the protein content in *M*. *officinalis* and total energy content in *T. pratense* (*P* < 0.05). On the other four host plants, the titer of *Buchnera* was correlated to biological characteristics and nutritional metabolism, but it has not reached a significant level. It suggests that the host plant influences the biological characteristics, nutrient metabolism and content of symbiotic bacteria in the pea aphid and that the titer of symbiotic bacteria in the pea aphid is actively regulated according to the nutrient status of the host plant.

**Table 1:** Correlation between the *Buchnera* titer and the biological characteristics and nutritional metabolism of pea aphids on different host plants.

Index	*M. officinalis*		*V. faba*		*P. sativum*		*T. pratense*		*M. sativa*		*O. viciaefolia*	
	Correlation coefficient	*P*	Correlation coefficient	*P*	Correlation coefficient	*P*	Correlation coefficient	*P*	Correlation coefficient	*P*	Correlation coefficient	*P*
Weight	-0.994	0.070	0.874	0.323	0.712	0.496	-0.698	0.508	0.966	0.167	-0.992	0.080
Fecundity	-0.989	0.095	-0.646	0.553	0.981	0.123	-0.733	0.476	-0.863	0.174	0.296	0.808
Protein	0.978*	0.022	-0.816	0.184	-0.900	0.100	0.033	0.967	0.270	0.730	-0.253	0.747
Soluble sugar	0.777	0.223	-0.325	0.675	-0.893	0.107	0.424	0.576	-0.913	0.087	0.778	0.222
Total lipids	-0.779	0.221	-0.285	0.715	-0.151	0.849	0.535	0.465	-0.267	0.733	-0.898	0.102
Glycogen	-0.669	0.331	0.366	0.634	0.853	0.147	0.561	0.439	0.075	0.925	-0.644	0.356
Total energy	0.892	0.108	-0.361	0.639	-0.883	0.117	0.981*	0.019	-0.949	0.051	0.753	0.247

* indicates significant relevancy at 0.05 level.

## 4 Discussion

Aphids use mouthparts to obtain nutrients from the phloem sieve elements of plants, but due to the absence of essential amino acids, the main source of nutrients does not satisfy the development and reproduction of aphids (Nalam et al., 2019). Symbiotic bacteria synthesize vitamins and other nutrients for the host insect, which affects the growth and development of the host insect. (Russell et al., 2014; Manzano-Marı et al., 2020). This study found that pea aphids differed in body weight and fecundity after feeding on different host plants, showing higher fecundity and weight gain on *V. faba*. This is similar to the findings of Han et al. (2012). This may be related to the differences in insect development, morphology and physiological characteristics caused by different host plants (Peccoud et al., 2010). This shows that feeding on *V. faba* is conducive to the growth and development of pea aphids. This also shows that different host plants have different effects on the growth of aphids, and *Buchnera* is very important for aphids to obtain nutrients on the host (Xu et al., 2023).

Symbiotic bacteria not only obtain and recover nitrogen-containing precursors from the food and nitrogenous metabolic substances of the host (Wang et al., 2020). It can also provide substances such as pectinase to help the host feed, facilitating the absorption and metabolism of nutrients by the host (Salem et al., 2017). Previous studies have found that the elimination of the *Buchnera* will reduce the protein contents of pea aphids, and the fecundity of pea aphids is in the direct ratio to its protein content: the higher the protein content, the higher the fecundity (Lv et al., 2018). So, the titer of the *Buchnera* was inextricably linked with the biological characteristics of the pea aphid. However, this experiment revealed found that the *Buchnera* titers of pea aphid on different hosts was different, among which the *Buchnera* titer on *T. pratense* was the highest. Thairu and Hansen (2019) that *Buchnera* sRNAs were differentially expressed when aphids fed on different plants. There are three reasons for this: firstly, Phytotoxins or secondary metabolites produced by different host plants can inhibit or promote the increase of the symbiotic bacteria titer in pea aphids, and secondary metabolites also have an inhibitory and bactericidal effect (Pawar et al., 2022). Secondly, significant differences in the amino acid composition of different host plants can interfere with the optimal symbiont density (Sandstrom and Pettersson, 1994; Douglas, 2003). Thirdly, aphids actively adjust their symbiotic density according to the nutritional status of the different host plants (Guilhot et al., 2020). Therefore, feeding on different host plants will directly affect the growth, development and nutritional metabolism of pea aphids. The results further verified the hypothesis that host plants will affect the symbiotic bacteria of phytophagous insects (Zhang et al., 2016).

Protein, carbohydrates, glycogen, and lipids, as the main forms of insects’ energy storage, have an important influence on the development and life activities of insects (Ahsaei et al., 2014). Saccharides and lipids are used as a source of energy for movement, providing energy for the host’s muscles as the insect walks or escapes (Hansford and Johnson, 1975; Martin and Lieb, 1979). Previous studies have demonstrated that eliminating *Buchnera* in pea aphid not only reduces the content of protein, but also slows down the feeding behavior of the pea aphid (Lv et al., 2018). This study found that differences in *Buchnera* levels in the pea aphid were due to feeding on different host plants, which affected the levels of saccharides and the insect’s behavior. It is indicated that *Buchnera* can also affect the feeding of pea aphids and their adaptability to host plants by participating in the interaction between plants and aphids (MaChado-Assefh et al., 2015; MaChado-Assefh and Alvarez, 2018). In addition, the total lipid and energy content of pea aphid are also different on different hosts, which may be because the saccharides in the aphid eventually store energy in the form of lipids (Febvay et al., 1999). Therefore, feeding on different hosts can lead to a difference in the *Buchnera* titer, showing the difference in the nutritional metabolism of pea aphids. However, in many insect systems, the density of obligate symbiosis is actively adjusted by the host to cope with the changes in environmental and physiological factors (Whittle et al., 2021). This experiment found that the correlation between the titer of *Buchnera* and the life activities and nutritional metabolism of pea aphids varied with host plants. This shows that insect hosts will actively adjust the symbiotic density according to the availability of nutrients and dietary requirements (Wilkinson et al., 2007; Snyder et al., 2012). Therefore, insect hosts benefit from symbiosis with obligate symbionts and have costs related to providing energy and nutrients to maintain the symbiotic population (Engl et al., 2020).

To summarize, host plants not only affect the biological characteristics and nutritional metabolism of pea aphids, but also regulated the symbiotic density. A previous study found that the density of *Buchnera* in cotton aphids differed among aphid populations fed on different host plants (Zhang et al., 2016). However, the titer of symbiotic bacteria is closely related to the nutritional metabolism of pea aphids, so different host plants will further influence the biological characteristics and nutritional metabolism of pea aphids by affecting the titer of symbiotic bacteria. This study demonstrates that among the six host plants, pea aphid has good adaptability on *V. faba*, and different host plants will also interfere with the nutritional function of *Buchnera*. These changes may lead to the transfer of energy level of pea aphids, thus affecting the growth and development of pea aphids. However, the changes in nutritional requirements of pea aphids fed on different host plants and the cost of symbiosis with obligate symbionts need further study. In addition, aphids with different phenotypes may be produced, accelerating the population evolution and species formation of aphids and responding to the changes in the external environment. Because of the geographical latitude difference, temperature and light work together in nature. Therefore, on the basis of this study, field experiments were carried out to explore the effects of the external natural environment on the growth, development and nutritional metabolism of aphids with different phenotypes and clarify the symbiotic-mediated population evolution and speciation of herbivorous insects. It provides a theoretical basis for further study on population evolution and species formation of pea aphid, and provides powerful information for designing better control strategies.

## Data availability statement

The original contributions presented in the study are included in the article/supplementary material. Further inquiries can be directed to the corresponding author.

## Author contributions

H-PL: Investigation, Formal analysis, Validation, Writing – original draft. Q-YY: Investigation, Data curation, Formal analysis, Writing – original draft. J-XL: Resources, Validation, Data curation. IH: Data curation, Writing – review & editing. YL: Formal analysis, Resources. Q-YZ: Resources, Data curation. KA: Writing – review & editing. AA: Writing – review & editing. C-ZL: Conceptualization, Resources, Supervision. NL: Conceptualization, Funding acquisition, Supervision, Writing – original draft, Writing – review & editing.
