# Supplementary material for: Host plant-mediated effects on Buchnera symbiont: implications for biological characteristics and nutritional metabolism of pea aphids (Acyrthosiphon pisum)
Source: Front Plant Sci. 2023 Dec 6;14:1288997. doi: 10.3389/fpls.2023.1288997 (PMC10731267; doi:10.3389/fpls.2023.1288997)
Supplement: Supplementary file 1 [file Table_1.docx]

Host Plant-Mediated Effects on *Buchnera* Symbiont: Implications for Biological Characteristics and Nutritional Metabolism of Pea Aphids (*Acyrthosiphon pisum*)

**Hui-Ping Liu^1^, Qiao-yan Yang^1^, Jing-xing Liu^1^, Inzamam Ul Haq^1^, Yan Li^1^, Qiang-yan Zhang^1^, Kotb A. Attia^2^, Asmaa M. Abushady^3,4^, Chang-zhong Liu^1^, Ning Lv^1^***

^1^Biocontrol Engineering Laboratory of Crop Diseases and Pests of Gansu Province, College of Plant Protection, Gansu Agricultural University, Lanzhou, 730070, China.

^2^Department of Biochemistry, College of Science, King Saud University, P.O. Box 2455, Riyadh 11451, Riyadh, Saudi Arabia.

^3^Biotechnology School, Nile University, 26th of July Corridor, Sheikh Zayed City, Giza, 12588, Egypt

^4^Department of Genetics, Agriculture College, Ain Shams University, Cairo, Egypt

*** Correspondence:** [lvn@gsau.edu.cn](mailto:lvn@gsau.edu.cn).

# Supplementary Information 1: Primer information

Table **S1** Primer information used for quantitative real-time PCR

| Gene | Spices | Primer | Primer sequence | References |
| --- | --- | --- | --- | --- |
| 16S rRNA | *Buchnera* | 16S rRNA-F | 5'-GGACCTTAAAAGGCCTCATGC-3' | Zhang et al., 2016 |
|  |  | 16S rRNA-R | 5'-GCTGGTTATCCTCTCAGACCAG-3' |  |
| ef1α | Aphid | ef1α-2F | 5'-CTTTCGTTCCCATCTCTGGATG-3' | Zhang et al., 2022 |
|  |  | ef1α-2R | 5'-CCGTCAGCCTTTCCTTCTTTACG-3' |  |
